# Compressive Behavior of 316L Stainless Steel Lattice Structures for Additive Manufacturing: Experimental Characterization and Numerical Modeling

**DOI:** 10.3390/biomimetics10100680

**Published:** 2025-10-10

**Authors:** Ignacio Ríos, Laurent Duchêne, Anne Marie Habraken, Angelo Oñate, Rodrigo Valle, Anne Mertens, César Garrido, Gonzalo Pincheira, Víctor Tuninetti

**Affiliations:** 1 Department of Mechanical Engineering, Universidad de La Frontera, Temuco 4811230, Chile; ignacio.rios@ufrontera.cl; 2 Master Program in Engineering Sciences, Faculty of Engineering, Universidad de La Frontera, Temuco 4811230, Chile; 3 Department ArGEnCo-MSM, University of Liège, 4000 Liège, Belgium; l.duchene@uliege.be (L.D.); anne.habraken@uliege.be (A.M.H.); 4 F.R.S.—FNRS, Rue d’Egmont 5, 1000 Bruxelles, Belgium; 5 Department of Materials Engineering (DIMAT), Faculty of Engineering, Universidad de Concepción, Concepción 4070138, Chile; aonates@udec.cl; 6 Construction Multidisciplinary Research Group, Facultad de Arquitectura, Construcción y Medio Ambiente, Universidad Autónoma de Chile, Talca 3460000, Chile; rodrigo.valle@uautonoma.cl; 7 Metallic Materials Science (MMS), University of Liège, Quartier Polytech 1 Allée de la Découverte 13 (B52), 4000 Liège, Belgium; anne.mertens@uliege.be; 8 Department of Mechanical Engineering, Universidad del Bío-Bío, Concepción 4081112, Chile; cgarrido@ubiobio.cl; 9 Department of Industrial Technologies, Faculty of Engineering, University of Talca, Camino a Los Niches Km 1, Curicó 3344158, Chile; gpincheira@utalca.cl

**Keywords:** body-centered cubic (BCC) lattice, selective laser melting (SLM), laser powder bed fusion (LPBF), stainless steel 316L, relative density, compression behavior, finite element analysis (FEA), energy absorption, mechanical metamaterials, biomedical implants

## Abstract

Lattice structures produced by additive manufacturing are increasingly used in lightweight, load-bearing applications, yet their mechanical performance is strongly influenced by geometry, process parameters, and boundary conditions. This study investigates the compressive behavior of body-centered cubic (BCC) 316L stainless steel lattices fabricated by laser powder bed fusion (LPBF). Four relative densities (20%, 40%, 60%, and 80%) were achieved by varying the strut diameter, and specimens were built in both vertical and horizontal orientations. Quasi-static compression tests characterized the elastic modulus, yield strength, energy absorption, and mean force, while finite element simulations reproduced the deformation and hardening behavior. The experimental results showed a direct correlation between density and mechanical properties, with vertically built specimens performing slightly better due to reduced processing defects. Simulations quantified the effect of strut–joint rounding and the need for multi-cell configurations to closely match the experimental curves. Regardless of the boundary conditions, for a density of 20%, simulating a single cell underestimated stiffness because of unconstrained strut buckling. For higher densities and thicker struts, this sensitivity to boundary conditions strongly decreased, indicating the possibility of using a single cell for shorter simulations—a point rarely discussed in the literature. Both experiments and simulations confirmed Gibson–Ashby scaling for elastic modulus and yield strength, while the tangent modulus was highly sensitive to boundary conditions. The combined experimental and numerical results provide a framework for the reliable modeling and design of metallic lattices for energy absorption, biomedical, and lightweight structural applications.

## 1. Introduction

Cellular materials, including foams, honeycombs, and lattices, are defined by their porous architectures. Such structures are not unique to human innovation; their principles are deeply embedded in natural systems. The lightweight yet robust framework of bone, the shock-absorbing design of a spider web, and the efficiency of a beehive’s honeycomb illustrate how ordered porosity can deliver mechanical advantages beyond those of bulk solids. Inspired by these biological examples, engineers have created synthetic cellular materials that combine high strength-to-weight ratios, efficient load transfer, and tailored energy absorption. With the development of additive manufacturing, particularly laser powder bed fusion (LPBF), it is now possible to fabricate these complex geometries with high precision, enabling their use in demanding fields such as aerospace, automotive, and biomedical engineering [1]. Within this broader class, lattice structures, distinguished by their intricate, repeating unit cells, have garnered significant attention across diverse engineering disciplines due to their exceptional strength-to-weight ratios and tunable mechanical properties [1,2,3,4,5]. These architected materials, particularly those with body-centered cubic configurations, offer enhanced mechanical attributes for applications ranging from aerospace and automotive to biomedical and blast protection [6]. The ability to tailor their macro-scale behavior through microstructural design makes them highly desirable for energy absorption applications, as demonstrated by their superior performance compared to uniform structures [7]. Moreover, the design of these structures can be optimized to improve compressive behavior and reduce stress concentration, thereby enhancing their overall performance and preventing premature failure [8]. Their mechanical behavior follows the scaling principles described by Gibson and Ashby, linking elastic and plastic properties to relative density [9,10]. These laws predict that cellular solids may be tuned for stiffness- or energy-optimized applications by controlling geometry and porosity. One notable example is the body-centered cubic (BCC) unit cell has emerged as a widely studied architecture due to its simple geometry, isotropic elastic response, and balance between bending- and stretching-dominated deformation [11,12]. The mechanical efficiency of BCC lattices has been demonstrated in both experimental and computational studies, which highlight their ability to sustain high specific stiffness and energy absorption relative to density [12,13]. Furthermore, BCC structures provide a useful baseline for exploring process–structure–property relationships in additively manufactured lattices [14,15,16].

Laser powder bed fusion (LPBF) enables the fabrication of complex lattice geometries in metals such as 316L stainless steel with high geometric fidelity [1,17]. However, LPBF introduces challenges including surface roughness, porosity, residual stresses, and dimensional deviations, which influence mechanical properties [18,19]. Build orientation is particularly important, as vertically fabricated struts typically display higher strength and stiffness than horizontal ones due to differences in melt pool stability and surface quality [20,21]. Understanding how these process-induced features interact with relative density is essential for reliable design. LPBF additive manufacturing enables the creation of materials with highly controlled microarchitecture. Mechanical evaluation of graded density lattice structures, such as Al-Si_14_-Mg and 316L stainless steel, has shown that energy absorption, stiffness, and yield strength are strongly influenced by the unit cell geometry and relative density [22,23]. Furthermore, research on micro lattice architecture has shown that geometric imperfections, surface roughness, and defects introduced during fabrication can substantially affect mechanical performance [24,25,26]. Understanding and mitigating these fabrication-induced variations is essential for reliable metal lattice design. Recent studies have also highlighted that the microstructural characteristics of LPBF 316L, including grain morphology and phase stability, directly affect tensile strength and ductility [27]. Other research has reported the influence of heat treatment and build orientation on the mechanical anisotropy and wear resistance of LPBF 316L [28,29]. Furthermore, tribological evaluations revealed that wear behavior strongly depends on process-induced surface characteristics and density gradients [30]. Complementary work emphasizes the need for optimized processing strategies such as hot isostatic pressing to mitigate porosity and enhance fatigue resistance [31]. Additional investigations into thermal history and scan strategy effects show that energy input conditions critically control defect formation and residual stress evolution in LPBF 316L [32]. Taken together, these contributions highlight that while 316L lattices fabricated with LPBF show significant potential, their mechanical reliability is still limited by processing-induced imperfections and microstructural variability.

On the other hand, the fluidity and packing of metal powders are fundamental to selective laser melting processes, as they determine the stability of the melting process, the uniformity of density, and the formation of defects [33]. Single-track and multi-track formation studies reveal that process parameters such as laser power, scanning speed, and grid spacing determine the quality and dimensional accuracy of the struts [34]. Post-processing and heat treatment of alloys such as Ti–6Al–4V have been shown to modify the microstructure, improving mechanical properties and enabling functional performance in critical applications [35]. In addition to microstructural control, energy absorption is another key property of lattices, particularly for crashworthiness and protective applications [21,36]. Prior studies on metallic foams and lattice structures have reported that energy absorption often peaks at intermediate densities due to early densification [32,37]. Ordered lattices such as BCC, however, tend to display monotonic increases in energy absorption with density, reflecting efficient load redistribution and stable deformation modes [38,39,40]. These characteristics have positioned lattices as promising alternatives to foams in applications requiring predictable mechanical response [41,42,43]. Beyond structural uses, metallic lattices have attracted growing interest for biomedical applications. Their interconnected porosity also supports osseointegration and vascularization, enhancing biological performance [44,45,46]. Studies have shown that LPBF lattices can be tailored to balance stiffness, permeability, and biofunctionality, making them suitable for orthopedic and dental implants [47,48,49]. For example, porous titanium scaffolds manufactured using additive manufacturing demonstrate favorable osseointegration and mechanical compatibility with bone tissue [46,50,51,52]. Computational shape optimization has been applied to femoral implants to improve structural performance, considering manufacturing constraints [53]. Experimental and numerical studies of 316L stainless steel lattice structures have validated their mechanical suitability for orthopedic and dental implants [54]. In order to optimize lattice performance in biomedical contexts, finite element modeling (FEM) provides a powerful tool for predicting lattice mechanics, enabling virtual testing of architectures and boundary conditions [55,56,57]. However, FEM models typically assume idealized geometries and constitutive laws, leading to discrepancies with experiments, particularly in yield strength predictions [55,56,57,58,59]. Boundary condition representation is also critical: single-cell models are efficient but may misrepresent deformation kinematics, while multi-cell models improve accuracy at higher computational cost [51]. As summarized by this review, in addition to small finite element size and the definition of a representative strut taking into account geometry defects, due to variation in the microstructures for struts with different orientations, their stress–strain curves might also not be identical. Systematic validation of FEM predictions against experimental results is therefore necessary to establish reliable design tools for LPBF lattices.

The present research measures the macroscopic anisotropy behavior for different densities providing interesting data for modelers. From the numerical point of view, a systematic analysis of the impact of finite element simulation boundary conditions of a single cell mesh shows inaccurate predictions of the plastic tangent modulus for a 20% density case. The final simulations also enhance that even with a refined multi-cell model, meshing round strut joints, the strut stress–strain behavior identified on LPBF bulk sample needs adaptation to predict the LPBF lattice experiments. Such a result for LPBF 316L material, found here by inverse modelling confirms the discussion of [60] where this issue was pointed both by crystal plasticity models and texture measurements. Given these considerations, this study investigates the compressive behavior of LPBF-fabricated 316L stainless steel BCC lattices with relative densities from 20% to 100%. Mechanical characterization through quasi-static compression testing is combined with finite element modeling under varied boundary conditions to evaluate scaling laws, orientation effects, and energy absorption capacity. The results deliver experimentally validated models that establish the design space of BCC lattices for lightweight structural, energy absorbing, and biomedical applications.

## 2. Materials and Methods

### 2.1. Specimen Design and Fabrication by Laser Powder Bed Fusion

**Figure 1:**
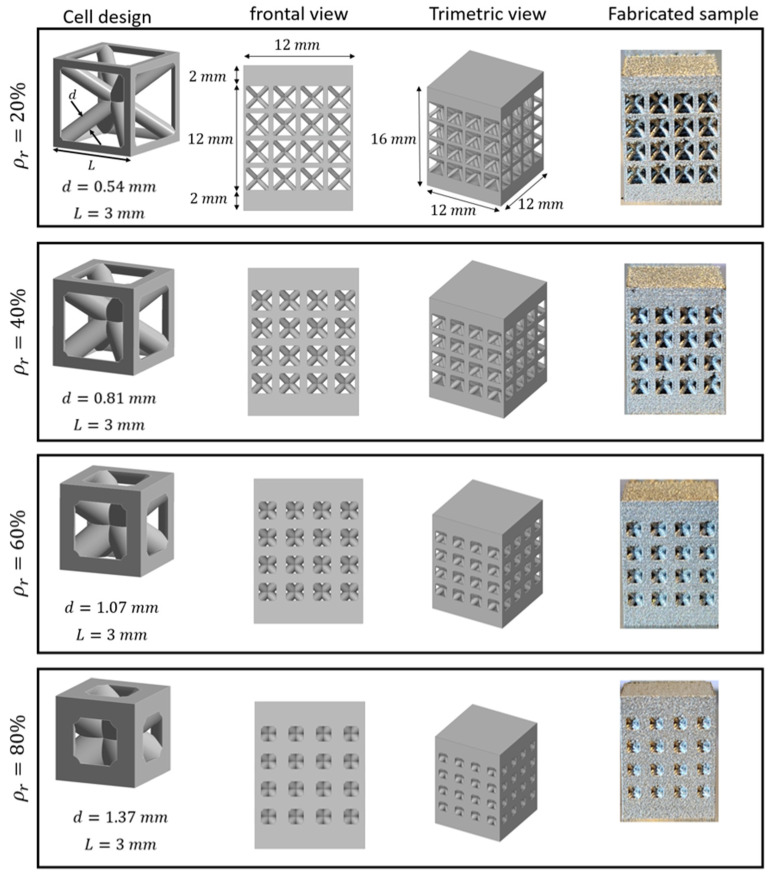
Design and fabrication of BCC lattice structures with four distinct relative densities: 20%, 40%, 60%, and 80%.

Body-Centered Cubic (BCC) lattice unit cell with struts reinforced on the x, y, and z axes was investigated in this research. The primary design parameters were the cell size (*L*) and the strut diameter (*d*). To evaluate the effect of material density on mechanical properties, the cell size was kept constant at 3 mm, while the strut diameter was varied to produce four distinct relative densities: 20%, 40%, 60%, and 80%. The corresponding diameters were 0.54, 0.81, 1.07, and 1.37 mm, respectively, and were estimated using a empirical relative density, in terms of diameters and unit cell size from *ρ*∗/*ρs* = 2.17(*d*/*L*) − 0.19, and the resulting designs were validated against CAD volume calculations to ensure accuracy before fabrication. These unit cells were arranged in a 4 × 4 × 4 matrix to form the final specimens, which had overall dimensions of 12 × 12 × 16 mm. A 2 mm solid bulk layer was added to the top and bottom of each specimen to simulate the solid outer walls of a typical component (Figure 1).

All specimens were fabricated using the Laser Powder Bed Fusion (LPBF) technique, specifically Selective Laser Melting (SLM), on an AconityMINI 3D printer (Aconity3D GmbH, Herzogenrath, Germany) using 316L stainless steel powder. The slicing and printing parameters were carefully controlled to ensure consistent results. Key settings included a layer thickness of 0.03 mm, a hatch distance of 0.08 mm, a laser power of 150 W, and a scanning speed of 900 mm/s. To assess the influence of part orientation, specimens were produced in two distinct build alignments: vertical and horizontal. The post-processing of horizontally built specimens, which required cutting the lattice structure directly from the build plate, slightly reduces the cross-section of external struts, which could slightly lower the mechanical properties compared to the vertical specimens. However, it is well-documented that horizontal struts exhibit lower geometrical accuracy with higher deviations from the nominal cross-section, therefore affecting the strength between vertical and horizontal build samples under compression [61,62].

### 2.2. Experimental Characterization of Mechanical Properties

**Figure 2:**
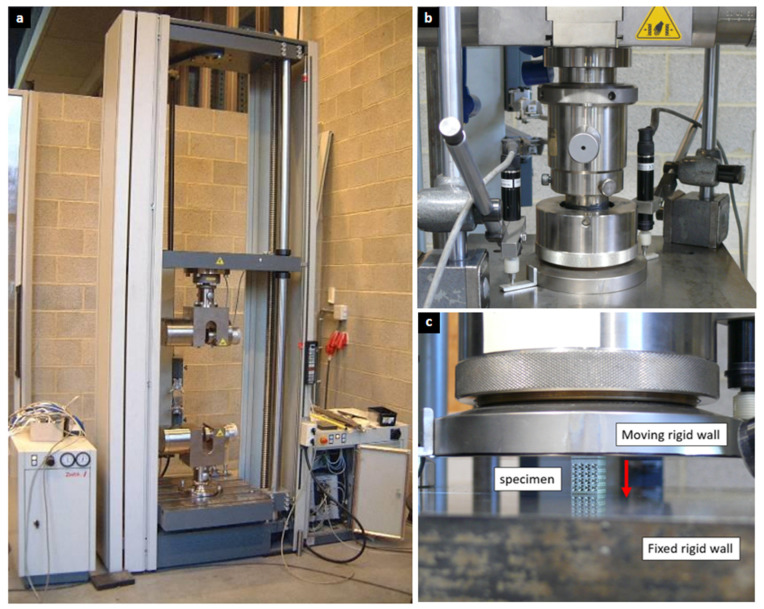
Experimental Methods for Compression Testing. (**a**) Zwick 100 kN universal testing machine used for the quasi-static compression tests on the fabricated specimens. (**b**) Depicts the experimental setup and loading conditions, (**c**) specimen positioned between the two rigid compressive plates.

Quasi-static compression tests were performed on all fabricated specimens using a Zwick 100 kN universal testing machine (ZwickRoell GmbH & Co. KG, Ulm, Germany) (Figure 2). The tests were designed to compress the specimens along their longitudinal axis, which aligned with the print direction for vertical samples and was orthogonal to it for horizontal samples. The protocol to maintain a nearly constant compressive strain rate and mitigate machine-compliance effects followed prior practices [63].

To characterize the mechanical response of the lattice structures, data processing involved converting the raw load–displacement data into apparent stress ($\sigma_{ap}$) and apparent strain ($\varepsilon_{ap}$) curves, treating the lattice as a homogeneous material. These parameters were calculated using Equation (1) and Equation (2), respectively.

(1) $$\sigma_{ap}=\frac{F_{i}}{A},$$

(2) $$\varepsilon_{ap}=\frac{u_{i}}{L},$$

where *F_i_* and *u_i_* are the instantaneous force and displacement, *A* is the cross-sectional area, and *L* is the original length of the specimen. The samples achieved a maximum strain of 10%; therefore, only the elastic and plateau regions were considered in this study, while densification and the associated failure mechanisms were not analyzed. From the resulting curves, total energy absorption capacity (TEA) (Equation (3)), and specific energy absorption (SEA) (Equation (4)) and mean force (*P_m_*) (Equation (5)) representing the average load-bearing capacity of the structure was computed.

(3) $$TEA=\int_{0}^{u}F_{i}dx,$$

(4) $$SEA=\frac{TEA}{M},$$

(5) $$P_{m}=\frac{TEA}{u},$$

u is the effective displacement at the point where the TEA is computed, and *M* is the mass of the structure.

### 2.3. Finite Element Modeling

**Figure 3:**
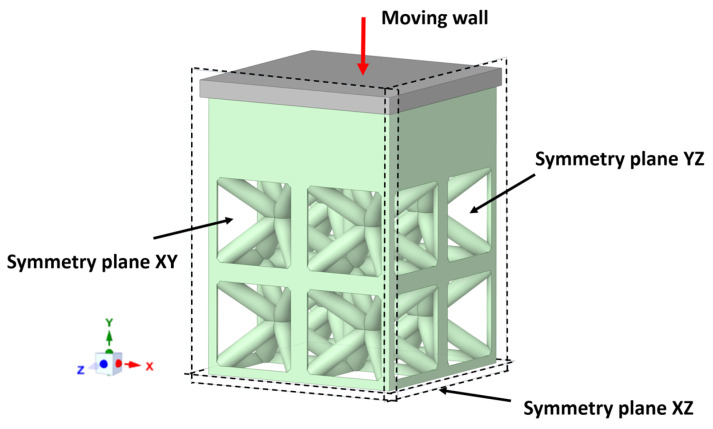
Numerical finite element (FE) model used for simulations. The 1/8 mesh model and the applied boundary and loading conditions of geometry’s symmetry.

Numerical simulations of the compression tests were performed using ANSYS Mechanical software to gain a deeper understanding of the deformation mechanisms and internal stress distribution. A bilinear elastic-plastic material model was utilized for 316L stainless steel, incorporating the strain-hardening behavior based on experimental data. The absence of significant nonlinear strain hardening and anisotropy along the studied ranged, the approximation of an isotropic yield surface and isotropic strain hardening of the material was used. The limitation of the stress-strain model adoptions that could influence the predictions is from the initial hardening zone, but this is generally considered negligeable for practical applications [64]. Additionally, the sample behavior confirms the isotropic initial yield surface assumption for the model for practical predictions of strength and energy absorption [60]. Elastic modulus *E* = 150 GPa, Poisson’s ratio *ν* = 0.3, yield strength *σ*_*y*_ = 225, and tangent modulus *H* = 0.95 GPa were obtained from experimental true stress–strain curves of fully dense printed sample. Struts were meshed with 10-node SOLID187 tetrahedral elements. Element sizes ranged from 0.15 to 0.24 mm, producing ~100,000 elements for the different density lattices models. Convergence was achieved when stiffness variation was below 5% between successive refinements. The bottom surface of the model was fully constrained, while the top surface was displaced vertically by a rigid platen. Contact was modeled as either frictionless or frictional (µ = 0.2 and 0.6). To optimize computational resources, a 1/8 mesh model was developed for each specimen, leveraging symmetry boundary conditions on the XY, XZ, and YZ planes (Figure 3).

### 2.4. Modeling Boundary Conditions and Size Effect

**Figure 4:**
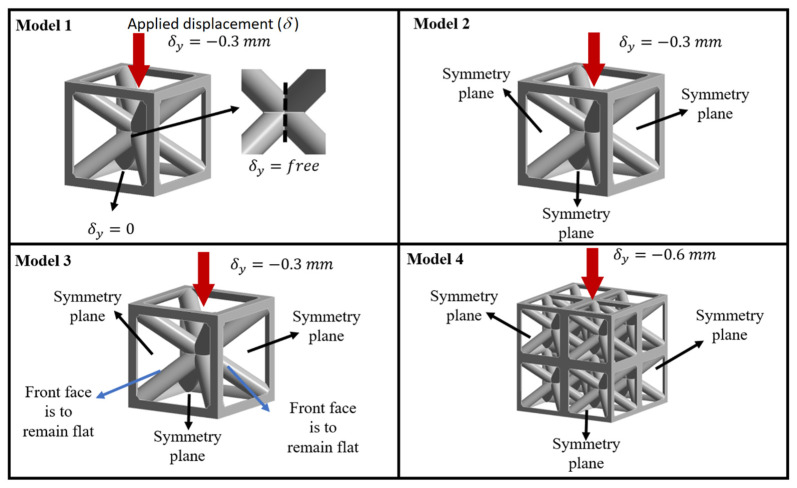
Four models with different boundary conditions. Models 1–3 are single-cell and Model 4 is multi-cell, 2 × 2 × 2.

To assess the influence of boundary conditions, four FEM models were created (Figure 4):

1. Model 1: Single cell with fixed base and free lateral faces.
2. Model 2: Single cell with three symmetry planes (equivalent to eight cells).
3. Model 3: Single cell with symmetry planes and coplanarity constraints (buckling suppressed).
4. Model 4: 2 × 2 × 2 multi-cell model with symmetry planes (equivalent to 64 cells). All models were compressed to 10% strain under displacement control.

In the single-cell configuration with a fixed base and free lateral faces (Model 1), the bottom face was fixed, while the top was displaced, and all lateral faces were free to deform. This lack of constraint made the vertical struts susceptible to buckling, representing the most unconstrained and least stiff boundary condition. It provided a lower-bound reference for the mechanical response of a single lattice cell.

In Model 2, the single-cell with three symmetry planes was constrained along three orthogonal directions, effectively reducing the problem to one-eighth of the geometry (equivalent to eight cells). This setup partially constrained deformation by enforcing mirror symmetry, thereby limiting lateral displacements compared to Model 1. It captured the behavior of a repeating unit within a periodic lattice structure.

In Model 3, the single cell with symmetry planes included additional coplanarity constraints. In this configuration, symmetry planes were applied along three orthogonal directions, while additional coplanarity constraints were imposed on the front and back faces to ensure they remained flat during loading. These constraints suppressed the out-of-plane deformation and buckling of the vertical struts observed in unconstrained models (e.g., Models 1 and 2), thereby representing an idealized case where lateral faces were perfectly supported. This configuration allowed the model to approximate the behavior of a cell embedded within a larger lattice, where surrounding cells provided constraint against buckling.

The last configuration, Model 4, was a 2 × 2 × 2 multi-cell model with symmetry planes. It consisted of a cube of 64 cells, with symmetry planes applied to reduce computational effort. Unlike the single-cell cases, this setup explicitly accounted for the interaction among multiple neighboring cells, distributing load and constraining lateral deformation. As a result, it more realistically represented the bulk response of a large lattice under compression.

## 3. Results

### 3.1. Experimental Characterization and Analytical Modeling

The experimental compression tests provided a comprehensive dataset on the mechanical behavior of the fabricated BCC lattice structures. Load–displacement and stress–strain curves generally exhibit the three characteristic deformation stages of cellular materials: an initial linear elastic region, a subsequent plastic deformation plateau, and a final stage of densification.

**Figure 5:**
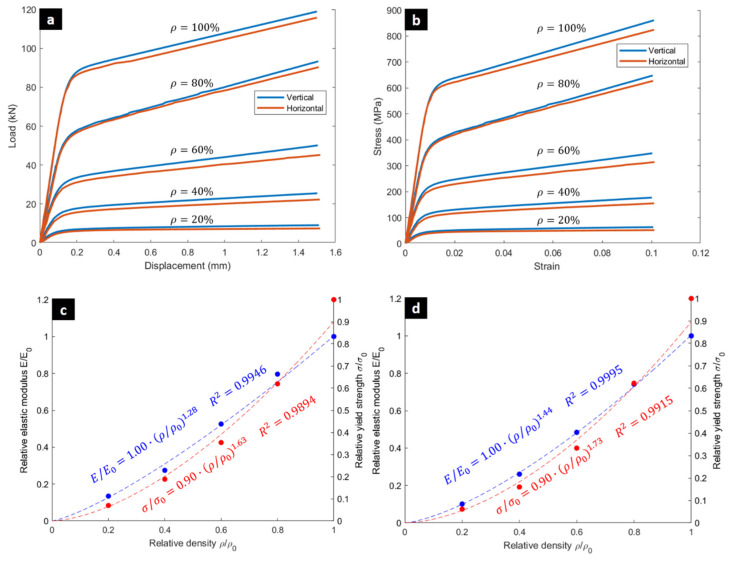
Experimental Compressive Behavior of 316L BCC Lattice Structures. (**a**) Load–displacement curves from the experimental compression tests for specimens with varying relative densities and build orientations. (**b**) Corresponding stress–strain curves showing the direct correlation between increased relative density and enhanced mechanical properties. (**c**) Gibson-Ashby model fit for the relative elastic modulus and yield strength of the vertically- and (**d**) horizontally oriented specimens (where *E*_0_ and σ_0_ are related to the bulk material).

Figure 5a presents the load–displacement curves for specimens with varying relative densities and build orientations. As expected, specimens with higher relative densities required greater force to deform, with the densest samples not reaching the densification stage due to the force limits of the testing machine. The corresponding stress–strain curves in Figure 5b show a proportional increase in elastic modulus and compressive strength with increasing relative density. A modest performance difference is observed between the two build orientations, which can be attributed to variations in melt pool stability, surface roughness, and defect distribution along the struts. Surface irregularities and partially melted particles, particularly on down-facing struts, act as local stress concentrators that promote early yielding and reduce stiffness—an effect consistent with previous findings on LPBF Ti-6Al-4V and Inconel lattices [62,65,66].

Table 1 summarizes the experimentally obtained mechanical properties, including elastic modulus, yield strength, tangent modulus, total and specific energy absorption, and mean force. The data reveal a near-linear increase in stiffness and strength with relative density, with vertical specimens consistently exhibiting slightly higher values—on average 8–12% higher modulus and 10–15% higher yield strength—compared to horizontally built counterparts. This limited anisotropy confirms that the chosen processing parameters ensured stable melt pool behavior and consistent interlayer bonding across orientations. The increasing trends in TEA and SEA also demonstrate efficient energy absorption with density, aligning with the power-law correlations in Figure 5c,d. In addition to TEA and SEA, other indicators such as the effective stroke ratio (ESR) can further characterize deformation efficiency and energy absorption performance [67,68].

The practical applicability of these results is that the obtained mechanical response of 316L BCC stainless steel lattices with relative densities between 20 and 40% achieves elastic moduli comparable to cortical bone (10–30 GPa), which may reduce stress shielding when used [50,51,52,53,54]. However, furter studies are required to confirm this by analyzing fatigue, osseointegration, and in vivo performance [69].

The experimental data also validates the Gibson-Ashby model (Equations (6) and (7)), confirming its accuracy in predicting mechanical properties for densities that were not physically tested.

(6) $$\frac{E}{E_{0}}=C{\left(\frac{\rho }{\rho_{0}}\right)}^{n}$$

(7) $$\frac{\sigma }{\sigma_{0}}=C{\left(\frac{\rho }{\rho_{0}}\right)}^{n}$$

where *E* and *σ* are the apparent elastic modulus and yield strength of the lattice structure, $E_{0}$ and $\sigma_{0}$ are the elastic modulus and yield strength of the bulk parent material. The fractions ($\frac{E}{E_{0}}$) and ($\frac{\sigma }{\sigma_{0}}$) define the relative modulus and the relative strength of the lattice structure, with C being the Gibson-Ashby constant. Its value depends on the cell topology and geometry and should be derived from experimental results. The exponent n depends on the mechanical response of the lattice structure, bending or stretch-dominated. It has different values depending on cell geometry size and materials. However, this exponent value is also usually derived from experimental results. Figure 5c,d illustrates the power-law fit of the experimental data for the vertical and horizontal specimens, which shows that the model accurately represents the relationship between relative density and both elastic modulus and yield stress.

### 3.2. Numerical Simulation and Size Effect Analysis

The numerical simulations provided a detailed explanation of the deformation mechanisms governing the behavior of the lattice structures. The study on size effect demonstrated that the structural response strongly depends on the number of unit cells included in the model. Simulations also confirmed that incorporating a rounding radius at the strut joints improves agreement with experiments, highlighting this as a critical factor for accurate modeling.

**Figure 6:**
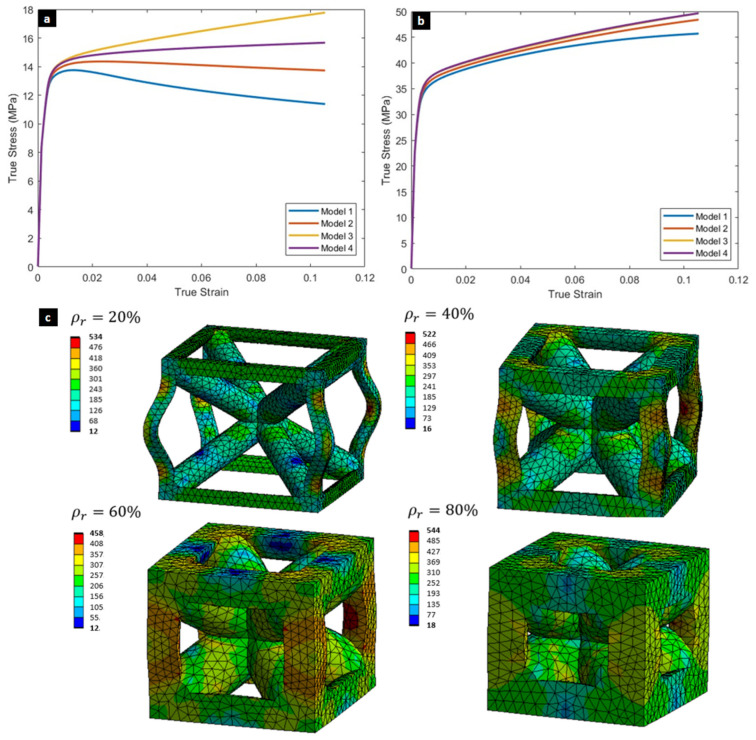
Numerical Simulation of the Size and Boundary Effect. The stress–strain curves for the four distinct boundary condition finite element models in (**a**) low-density (20% relative density) and (**b**) mid-density (40% relative density) lattice structures. (**c**) Deformed shape of a single cell (Model 1) with von Mises stress contours at a global strain of 10%, demonstrating the more pronounced buckling of external struts in smaller, unconstrained models.

For 20% relative density structures (Figure 6a), large differences in mechanical properties were observed, particularly in the plastic zone. Models 1 and 2 (single-cell and 8-cell unconstrained) showed softening after yielding due to buckling of the exterior vertical struts, as confirmed in the deformed shape of Model 1 (Figure 6c). In contrast, Models 3 and 4 (constrained 8-cell and 64-cell) exhibited greater stiffness and avoided this softening. This indicates that simplified single-cell models, though computationally efficient, are inadequate for capturing the true behavior of embedded lattices.

At 40% relative density (Figure 6b), no softening was observed, though differences in tangent modulus remained, with variations exceeding 200% for the 20% relative density models (Table 2). The tangent modulus proved the most sensitive property to boundary conditions and cell count, as thin outer struts in low-density lattices are more prone to buckling, a phenomenon reduced in multi-cell arrangements with greater constraint.

In summary, the simulations demonstrate that size effect is a critical factor, especially for low-density lattices. Tangent modulus is the most affected property, emphasizing that single-cell models are insufficient proxies for predicting the behavior of larger, embedded structures.

**Figure 7:**
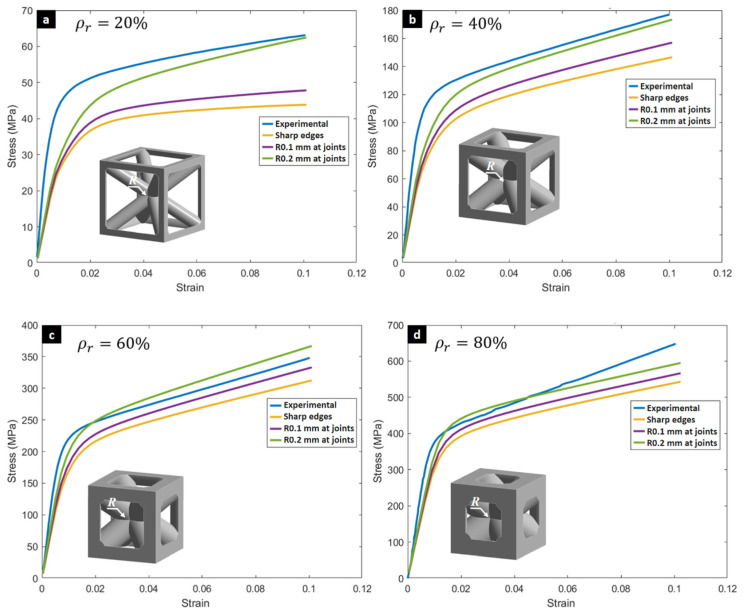
Validation of Analytical and Numerical Models. Comparative stress–strain curves of experimental results and numerical simulations for specimens at (**a**) 20%, (**b**) 40%, (**c**) 60%, and (**d**) 80% relative density, demonstrating consistency in overall trends and the effect of incorporating a rounding radius at strut joints in the simulations.

The final validation of the numerical model was achieved by comparing the simulated stress–strain curves with the experimental data, as shown in Figure 7. The simulations that incorporated a rounding radius at the strut joints correlated more closely with experimental results, suggesting that this manufacturing reality—the inherent rounding of deposited material at strut intersections—is a critical factor for accurate modeling.

**Figure 8:**
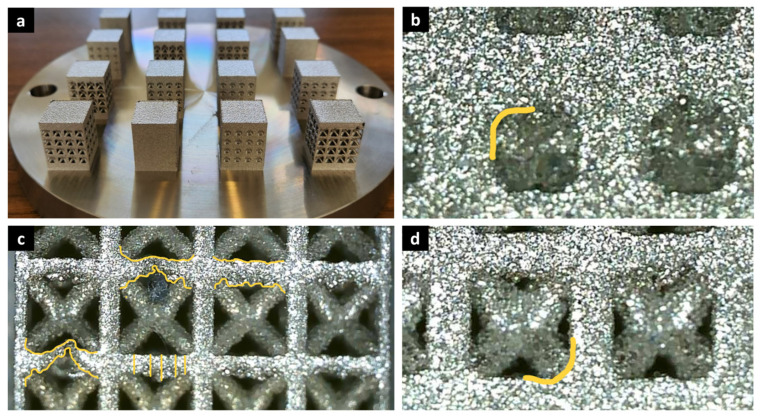
Representative images of LPBF-fabricated 316L BCC lattice specimens at different relative densities showing typical surface and geometrical imperfections. (**a**) Overview of printed samples on the build plate; (**b**–**d**) details with yellow lines highlighting surface roughness, strut waviness, and partially melted particles.

In addition to the validation of numerical results, the surface morphology and geometric imperfections of the as-built specimens were examined to better interpret the discrepancies between experiments and simulations. Figure 8 presents representative images of the manufactured BCC lattices at different relative densities. These observations reveal typical LPBF-induced surface features such as partially melted particles, strut waviness, and local porosity, particularly evident along down-facing surfaces and overhanging struts. The extent of these imperfections increases at lower relative densities (20–40%), where slender struts are more susceptible to melt pool instability and powder adhesion. Such features not only modify the effective cross-sectional area but also act as local stress concentrators that promote premature yielding and buckling. In contrast, higher-density specimens (60–80%) exhibit smoother surfaces and more uniform strut junctions, resulting in improved stiffness and strength. The incorporation of these geometrical realities into future FEM models—such as through stochastic perturbations of strut geometry or surface roughness fields—could further enhance predictive accuracy, especially in the low-density regime where deformation is bending-dominated.

Process–structure–property considerations further contextualize the observed deviations at low relative density. In metal AM, spatial variations in thermal history and cooling rate are known to drive melt-pool size fluctuations and microstructural heterogeneity, which in turn amplify geometric defects (roughness, strut waviness, locally reduced cross-section) and promote early buckling. Finite-element thermal modeling of directed energy deposition has shown that stabilizing the melt pool through appropriate power control reduces these gradients [70], and subsequent optimization work demonstrated that variable-power strategies can homogenize thermal fields and improve hardness uniformity—serving as a proxy for more uniform microstructure [71]. Although these studies address DED and AISI M4 steel, the mechanism—cooling-rate-controlled microstructure feeding back into geometric quality and mechanical response—is directly relevant to LPBF lattices. Together, these results support our interpretation that manufacturing-induced heterogeneity in thermal history underlies the surface morphology and effective-section deviations responsible for the larger model–experiment gap at 20% relative density.

**Figure 9:**
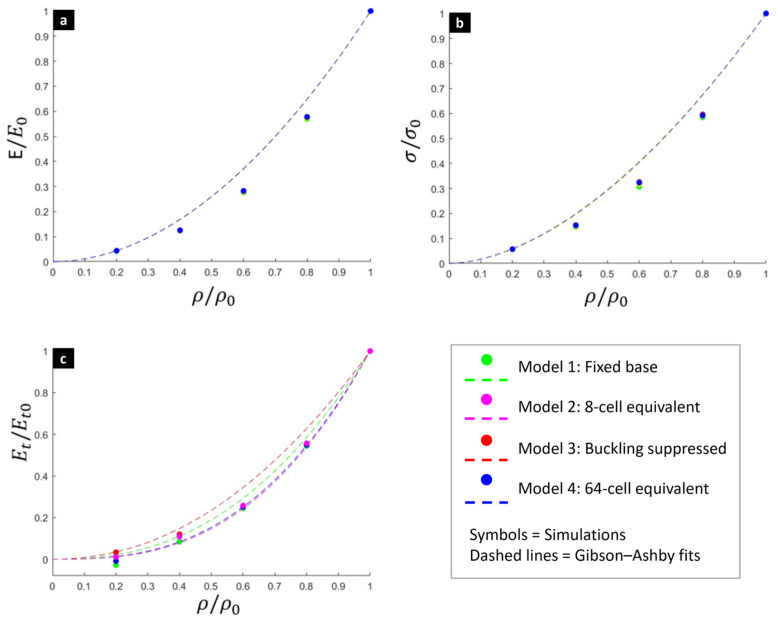
Gibson-Ashby model fit incorporating data from all four numerical models for (**a**) the relative elastic modulus, (**b**) yield strength, and (**c**) tangent modulus.

The Gibson-Ashby model also applied to the numerical data (Figure 9) from all four size effect models (1–4) confirms that the power function with *C* = 1 predicts the three relative mechanical properties as a function of the relative density. Elastic modulus the yield strength is predicted with good precision and low sensitivity to boundary conditions. However, differences emerge in the tangent modulus, where some variations in the exponent across the different models demonstrate the tangent modulus is highly sensitive to the size and boundary effects observed in the simulations. Thin outer struts in these low-density models are more susceptible to buckling, a phenomenon that is less pronounced in multi-cell arrangements where the struts are constrained by their neighbors. The Gibson-Ashby curves (Figure 9c) further illustrate this point, showing a significant change in the exponent for the tangent modulus across the different boundary condition models, while the curves for elastic modulus and yield strength (Figure 9a,b) nearly overlap, demonstrating their low sensitivity to the size effect.

## 4. Conclusions

This study investigated the compressive response of body-centered cubic (BCC) lattice structures fabricated from 316L stainless steel by laser powder bed fusion across relative densities ranging from 20% to fully dense. Quasi-static compression tests combined with finite element modeling provided a detailed understanding of the role of relative density, build orientation, and boundary condition assumptions. The results demonstrated that stiffness, yield strength, and energy absorption increased systematically with relative density. For instance, specimens with a relative density of 20% had an elastic modulus of 8.803 GPa, while those with a relative density of 80% reached 52.115 GPa. The specific energy absorption also increased with density, from 1.6067 J/g for a density of 20% to 8.5091 J/g for a density of 100%. At low densities, deformation was dominated by strut buckling, while at intermediate and high densities stable plastic struts were observed. The effect of build orientation on mechanical response was limited. Vertically fabricated specimens showed slightly higher stiffness and strength than horizontal ones, reflecting modest anisotropy in the layer-by-layer process. Differences in elastic modulus and yield strength generally remained within ~10–15% and dropped below 3% at higher densities, indicating that the chosen processing parameters minimized orientation-dependent behavior. Finite element simulations reproduced the experimental behavior with good accuracy when symmetry constraints and multi-cell configurations were included. Predictions of yield strength were within 8–12% of experiments, validating the approach for design purposes. Discrepancies were attributed to as-built imperfections not captured in the idealized models. The numerical simulations also provided a crucial understanding of the size effect, demonstrating that the mechanical response of a lattice structure is highly dependent on the number of unit cells it contains. For instance, the tangent modulus for a 20% relative density model varied by over 200% between different boundary condition models. It was found that a single-cell model is an inaccurate representation of a larger embedded lattice, as it fails to account for the crucial strut buckling and constraint effects that become apparent in multi-cell arrays. The tangent modulus was identified as the mechanical property most sensitive to this effect, highlighting the importance of using high-fidelity models for accurate performance prediction.

The findings highlight the tunability of BCC lattices. At lower densities, they are well-suited for crashworthiness applications, while at higher densities they approach bulk-like strength and stiffness for structural reinforcement. At intermediate densities, their modulus overlaps with cortical bone, suggesting potential for biomedical use. Nonetheless, claims of implant applicability should be substantiated by future investigations addressing fatigue behavior, osseointegration, and in vivo validation. Moreover, as the present study was limited to 10% strain, only the elastic and plateau regions were assessed, while densification and associated failure mechanisms were not captured. Extending the analysis to larger strains will be essential to fully understand the evolution of failure modes and the ultimate energy absorption capacity.

## Figures and Tables

**Table 1:** Experimental Mechanical Properties of 316L BCC Lattice Specimens.

Specimen	Elastic Modulus (GPa)	Yield Strength (MPa)	Tangent Modulus (GPa)	Total Energy Absorption, TEA (J)	Specific Energy Absorption, SEA (J/g)	Mean Force, $P_{m}$ (kN)
BCC-V-20	8.803	40.36	0.122	11.84	1.607	7.894
BCC-V-40	17.96	109.1	0.545	31.13	3.103	20.75
BCC-V-60	34.41	205.1	1.123	59.84	4.738	39.89
BCC-V-80	52.12	358.8	2.711	107.2	6.701	71.47
BCC-V-100	65.49	579.3	2.775	146.5	8.509	97.68
BCC-H-20	6.44	35.7	0.059	10.02	1.209	6.68
BCC-H-40	16.66	93.3	0.435	27.70	2.536	18.46
BCC-H-60	30.95	194.7	0.941	55.48	4.125	37.00
BCC-H-80	47.40	363.7	2.431	105.0	6.210	70.00
BCC-H-100	63.94	584.5	2.488	142.7	7.842	95.16

**Table 2:** Mechanical Properties of Simulated Lattice Structures.

Relative Density	Boundary Condition	Elastic Modulus (GPa)	Yield Strength (MPa)
	Model 1	6.433	12.6
20%	Model 2	6.487	12.8
	Model 3	6.546	13
	Model 4	6.526	13
	Model 1	18.56	33.2
40%	Model 2	18.68	33.9
	Model 3	18.79	34.7
	Model 4	18.80	34.5
	Model 1	41.71	69.6
60%	Model 2	42.02	71.7
	Model 3	42.31	73.8
	Model 4	42.36	73.3
	Model 1	85.60	132.6
80%	Model 2	86.06	133.9
	Model 3	86.55	135.1
	Model 4	86.67	134.2
100%	(Bulk material)	14.94	226.7

## Data Availability

The original contributions presented in this study are included in the article. Further inquiries can be directed to the corresponding author.
